# Mobile Phone–Based Personalized and Interactive Augmented Reality Pictorial Health Warnings for Enhancing a Brief Advice Model for Smoking Cessation: Pilot Randomized Controlled Trial

**DOI:** 10.2196/52893

**Published:** 2024-08-01

**Authors:** Ziqiu Guo, Yongda Wu, Man Ping Wang

**Affiliations:** 1 School of Nursing The University of Hong Kong Hong Kong China (Hong Kong); 2 Children's Hospital of Eastern Ontario Research Institute Ottawa, ON Canada

**Keywords:** augmented reality, mobile-based, smoking cessation, brief advice, pictorial health warning, pilot randomized controlled trial

## Abstract

**Background:**

Augmented reality (AR) is a novel modality for promoting smoking cessation (SC). AR-visualized adverse consequences for education and smoking prevention have only been evaluated in nonsmokers in previous studies.

**Objective:**

To assess the feasibility and preliminary effectiveness for SC of AR pictorial health warnings (PHWs) on cigarette packs.

**Methods:**

We conducted a pilot randomized controlled trial in adult daily smokers in communities in Hong Kong. All participants received AWARD (ask, warn, advise, referral, do-it-again) model–guided SC advice, a warning leaflet, and referral to SC services at baseline. Interactive, chat-based SC support comprising regular messages and real-time support was provided to all participants via instant messaging apps (eg, WhatsApp) for 3 months after randomization. Participants in the intervention group additionally received 6 links to the AR PHWs showing the worsening health status of various organs caused by smoking. The level of the AR PHWs was adjustable to smoking behaviors (ie, smoking duration or daily cigarette consumption) to increase interaction. Participants could swipe, drag, or rotate the 3D PHWs to reinforce their impression of the health consequences of smoking. The primary outcome was self-reported past 7-day point-prevalence abstinence (PPA) at 3 months. The acceptability of the AR intervention was assessed by the proportion of participants who had viewed AR PHWs during the intervention. Participants who viewed AR PHWs further evaluated the perceived effect of the AR PHWs on a scale of 0 (not helpful at all) to 10 (very helpful). Intention to treat was used, and the risk ratio (RR) of the intervention effect was estimated by Poisson regression.

**Results:**

From April to November 2021, 80 participants were recruited and randomly assigned to intervention (n=40) and control (n=40) groups. Most participants were male (66/80, 83%) and planned to quit beyond 30 days or were undecided (65/80, 81%). The intervention group had a higher but nonsignificant 7-day PPA (7/40, 18% vs 5/40, 13%; RR 1.40, 95% CI 0.48-4.07) and quit attempts (15/40, 38% vs 11/40, 28%; RR 1.36, 95% CI 0.71-2.60) at 3 months than the control group. In the intervention group, 17 of 40 (43%) participants viewed the AR PHWs. The AR PHWs had modest effects on knowledge of the adverse consequences of smoking on personal health (mean score 3.94, SD 3.52), reducing the frequency of buying cigarettes (mean score 3.29, SD 3.08), increasing the perceived importance of quitting (mean score 3.88, SD 3.50), and making the PHWs more disgusting (mean score 3.41, SD 3.08) and horrible (mean score 3.38, SD 3.05). The 3-month self-reported 7-day PPA was higher in those who ever (vs never) viewed the AR PHWs (5/17, 29% vs 2/23, 9%).

**Conclusions:**

The mobile-based interactive AR PHWs were feasible, and the effectiveness on smoking abstinence warrants further testing.

**Trial Registration:**

ClinicalTrials.gov NCT04830072; https://clinicaltrials.gov/study/NCT04830072

## Introduction

Brief advice is effective in promoting smoking cessation (SC) [1-4]. Pictorial and text warnings on adverse smoking consequences are always used to warn smokers about the harms of continued smoking when delivering brief SC advice. Pictorial warnings evoke negative emotions about smoking [5] and increase quit attempts [6] and quit intentions [5]. However, the effects of pictorial and text warnings can be attenuated, since some smokers feel disconnected from the smoking-attributable diseases being shown [7,8]. Enhancing pictorial and text warnings is needed to strengthen their effect on promoting SC. Augmented reality (AR) technology, allowing interaction using vivid 3D visual imaging, has potential for promoting SC and could be promising.

A recent experimental study found that AR-visualized messages about adverse consequences of smoking increased negative emotions toward smoking and willingness to engage in an SC campaign in college students [9]. AR-simulated adverse consequences of smoking (eg, in the lungs) were used in educating nonsmokers about smoking hazards to the human body [10] and dissuading nonsmokers from smoking [11], but the effects were not assessed using a rigorous research design. Recent studies have suggested the potential of AR as a novel modality for conducting cue exposure therapy for SC (ie, extinguishing cue-provoked urges to smoke by exposure to smoking-related cues) since smoking-related AR images were found to have a similar effect on eliciting cue-provoked urges to smoke compared to in vivo smoking cues [12,13]. Our literature review of PubMed (up to September 2023) using the terms “augmented reality” and “smoking cessation” found no study assessing the effect of AR visualization of adverse consequences from smoking on SC. Therefore, a pilot study is needed to assess the feasibility of AR visualization of adverse consequences from smoking for SC to inform a larger-scale, full trial.

Hong Kong has a relatively low smoking prevalence (9.5% in 2021 [14]). However, significant increases in the proportion of smokers without an intention to quit (12.7% to 69%) or past quit attempts (74.4% to 80.4%) were observed from 2009 to 2018 [15]. The penetration of mobile phones in Hong Kong has provided a novel avenue for SC, considering that less-motivated smokers might be receptive to mobile-based intervention [16]. Several studies have shown that widely used mobile instant messaging (IM) apps (eg, WhatsApp, WeChat) are a promising modality for delivering personalized and interactive SC interventions [2,17,18]. Communicating smoking-related adverse consequences with texts or pictures via IM apps was also found to be feasible [18]. These findings suggest that IM apps might be usable to deliver AR for smokers, especially smokers with a lower quit intention who are less willing to download and use additional SC-related apps [19]. Pictorial health warnings (PHWs) on cigarette packs portraying the adverse consequences of smoking and disseminating messages on quitting are widely and frequently seen by smokers (126 countries and jurisdictions finalized requirements for PHWs by 2021 [20]). Hong Kong introduced PHWs in 2007, and adopted 12 new PHWs with at least 85% coverage of the principal packaging of cigarettes in 2018. In Hong Kong, 88.6% of current smokers reported having seen PHWs in the past 30 days in 2018 [21]. This study used the notion that PHWs are widely viewed by the public on cigarette packaging and developed 6 new, localized, AR-based PHWs (Multimedia Appendix 1). Taking advantage of IM apps, this pilot study aimed to examine the feasibility and preliminary effect of AR PHWs on SC.

## Methods

### Ethical Considerations

Ethical approval was obtained from the institutional review board of the University of Hong Kong/Hospital Authority Hong Kong West Cluster (UW 21-018), and the trial was registered with ClinicalTrials.gov (NCT04830072). Written consent was obtained from all participants after informing them that they had the right to withdraw from the study at any time, and all the information collected was anonymized and was only used for research. After completing each follow-up, all participants were compensated with HK$ 50 (US$ 6.40). The Consolidated Standards of Reporting Trials (CONSORT) reporting guidelines and their extension to randomized pilot and feasibility trials were followed [22].

### Study Design, Setting, and Participants

We conducted a parallel, 2-arm, pilot randomized controlled trial (RCT) in Hong Kong. From April to November 2021, participants were individually recruited at smoking hot spots (ie, places where smokers linger and smoke) in community sites. Research staff proactively approached smokers using a “foot-in-the-door” technique, which is commonly used in SC trials in the context of smokers with low intention to quit [1,2,23] Briefly, the research staff initiated a conversation by asking about small and simple questions like their daily cigarette consumption and quit attempts. Those willing to talk were warned about the risk of continuous smoking and advised to quit smoking as early as possible. Smokers who expressed interest in SC were briefly introduced to the trial, screened for eligibility, and invited to join. Eligibility criteria included being Chinese, being a smoker, being aged 18 years or older, smoking at least 1 cigarette daily in the past 30 days, owning a smartphone with IM apps (eg, WhatsApp), and being able to communicate in Chinese. We excluded smokers who were participating in other SC services or projects.

### Randomization and Blinding

Participants were individually randomized to intervention or control groups (at a 1:1 ratio) according to a predefined randomization list with permuted block sizes of 2, 4, and 6. The allocation sequence was generated by a noninvestigator and was concealed from the research staff involved in participant recruitment. To avoid treatment contamination, we only recruited 1 smoker at 1 smoking hot spot at 10-minute intervals. Blinding of participants and the SC advisor who delivered the interventions was not possible. All outcome assessors and statistical analysts were blinded to treatment allocation until the primary analyses were completed.

### Interventions

All participants received brief SC advice following the AWARD (ask, warn, advise, referral, do-it-again) model [1-3] and were offered referrals to SC services at baseline. As a widely adopted alternative practice for smokers who are less ready to quit in SC programs [2,24], chat-based SC support was delivered to all participants for 3 months via IM apps (ie, WhatsApp). Guided by motivational interviewing (MI) techniques [25] and the transtheoretical model [26], the chat-based SC support included prescheduled messages and real-time support personalized to the quitting process provided by 1 SC advisor (a registered nurse trained in SC treatments and MI techniques). The 24 prescheduled messages’ content and frequency were adjusted according to participants’ willingness to quit at baseline based on the transtheoretical model (willing to quit within 30 days/after 30 days or undecided) [26]. For participants willing to quit within 30 days, the messages covered quitting strategies, coping skills, and encouragement. Participants who were not ready to quit within 30 days received messages on motivational advice, benefits of SC, hazards of smoking (and secondhand smoke), and encouragement. At least 1 message was delivered per week to initiate the conversation between participants and the SC advisor. The frequency of the prescheduled messages increased to 2 to 3 per week when approaching the quit date set by the participant. The real-time support was also personalized to participants’ inquiries and followed MI techniques to enhance motivation and self-efficacy of quitting. The SC advisor discussed the supporting content with experts in the SC team when necessary.

Six web AR links were integrated into the messages during the chat-based SC support for the intervention group. The AR links were built based on 6 of 12 local PHWs (due to the limited budget; Multimedia Appendix 1): “smoking takes away my voice,” “smoking causes “lung cancer,” “smoking causes peripheral vascular disease,” “smoking causes impotence,” “smoking causes aging,” and “smoking offence: fixed penalty HK $1500.” These 6 PHWs were chosen because they were particularly suitable for using 3D animations to show the disease’s progression and the increasing cost of smoking. One AR technology company developed the AR links with the SC experts’ continuous comments on the shape and color of the models and necessary changes. All the AR PHWs were designed to increase the participants’ awareness of the devastating effects on health of smoking and its ever-growing monetary cost, thereby promoting their engagement in the chat-based SC support. The AR PHWs could be accessed by using the smartphone’s camera and the mobile web browser on the 2 most widely adopted mobile operating systems, iOS and Android, without the requirement to download any apps.

**Figure 1:**
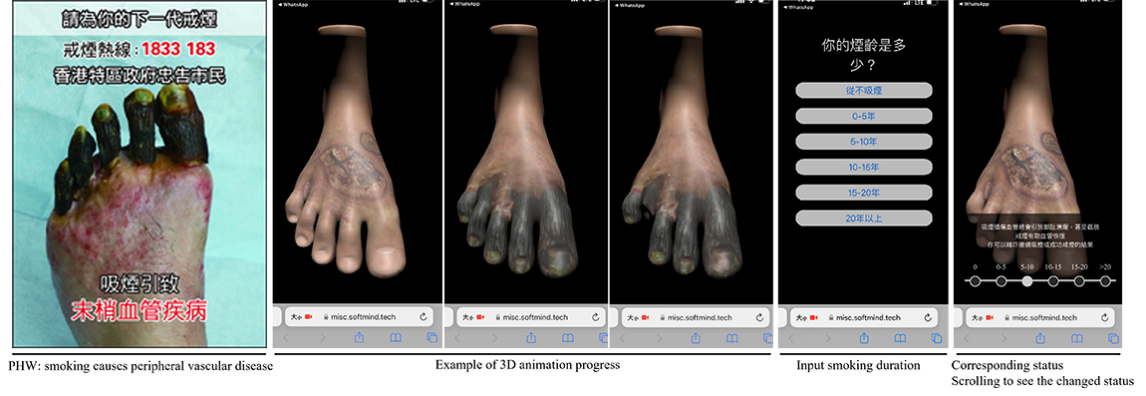
Example of pictorial health warning (PHW) and corresponding augmented reality PHW.

The web AR links were opened in a mobile web browser, and the participants were instructed to scan the corresponding PHWs to activate the AR links. Upon successfully scanning the PHWs, they were shown jointly with the corresponding augmented components (ie, 3D PHWs and texts) on the screen. Participants could interact with the augmented components and real-world images at the same time. By default, 30-second 3D animations of the PHWs were played to show the gradually worsening health status of organs (eg, changes in the color and shape of the lungs) because of continuous smoking. The corresponding health status of the organs was displayed in 3D when inputting smoking behaviors (ie, smoking duration or daily cigarette consumption). The 3D PHWs showing the status of the organs could be swiped, dragged, or rotated to be viewed from different angles. Participants could also interact with the 3D PHWs by dragging the horizontal scroll bar to indicate different durations of smoking (or levels of cigarette consumption) to see the changing status of the organs (the AR PHW for “smoking causes peripheral vascular disease” is shown in Figure 1). The original PHW for “smoking offence: fixed penalty HK $1500” used images of money to warn smokers that smoking in smoke-free areas has a fine of HK $1500 (US $192.30). To keep consistency with other AR PHWs that show changes in organs due to smoking, we redesigned this PHW to show the amount of money spent yearly based on daily cigarette consumption and the average price of a pack of cigarettes (HK$ 60; US $7.70) at the time of the study. When activated, the animation showed an increasing amount of money being burned due to smoking. The feeling of holding the 3D PHWs in their hands and viewing them from different angles might remind participants of the corresponding PHWs naturally and intuitively. The SC advisor also encouraged participants to click the links and interact with the AR PHWs and supplemented the information (eg, with corresponding PHWs or a brief introduction) as appropriate. As an alternative way to access the AR PHWs, we provided the PHW images on a separate device (eg, a personal computer running WhatsApp) so that the users could scan the images with their mobile phones.

### Measures

At baseline, we collected sociodemographic characteristics (sex, age, education, monthly household income, and employment status), smoking and quitting behaviors (daily cigarette consumption, nicotine dependency level [27], past quit attempts, willingness to quit, and self-efficacy [2]). The nicotine dependency level was measured by the Heaviness of Smoking Index (HSI), which sums up the scores of 2 questions: the number of cigarettes smoked per day and the time to the first cigarette after waking. Total HSI scores of ≤2, 3-4, and 5-6 indicate low, moderate, and high levels of nicotine dependency, respectively. The willingness to quit was measured by asking participants their planned quit date with responses of “within 7 days/30 days/60 days/undecided.”

Telephone follow-ups were conducted at 1 month and 3 months after intervention initiation. The primary outcome was self-reported 7-day point-prevalence abstinence (PPA) at the 3-month follow-up. Secondary outcomes included the 7-day PPA at the 1-month follow-up, smoking reduction by at least 50% of baseline daily cigarette consumption (excluding quitters), and quit attempts (defined by abstinence for at least 24 hours) at the 1- and 3-month follow-ups.

Considering that proactive recruitment and chat-based support via IM apps have shown feasibility in our previous SC studies [1,2,17,28], we calculated the proportion of participants who had viewed the AR PHWs during the intervention to assess the acceptability of the AR intervention component. The proportion was defined as the actual number of participants who reported having ever viewed the AR PHWs at the follow-ups divided by the number of participants in the intervention group. Missing values were treated as “no viewing.” In participants who viewed the AR PHWs, we further evaluated the perceived effect of the AR PHWs on (1) knowing the adverse consequences of smoking on personal health, (2) increasing the perceived importance of quitting, (3) reducing the frequency of buying cigarettes, (4) perceiving PHWs as more disgusting, and (5) perceiving PHWs as more horrible, each on a scale of 0 (not helpful at all) to 10 (very helpful).

### Sample Size Calculation

No formal sample size calculation was conducted. The sample size of 80 participants is comparable with previous studies of virtual reality interventions (ranging from 8-102 [29]) for SC and was deemed sufficient to test the feasibility and estimate the parameters to inform the design of a definitive RCT.

### Data Analyses

We compared the primary and secondary outcomes between the 2 groups using Poisson regression with robust variance [30], which yielded risk ratios (RRs) of the intervention effect. The model was further adjusted for imbalanced baseline characteristics (ie, education) as a sensitivity analysis. The intention to treat approach was adopted, assuming participants lost to follow-up did not change their smoking behaviors (eg, they made no quit attempts nor changed their daily cigarette consumption) [31]. The acceptability and perceptions of the AR PHWs were reported descriptively using the proportion of participants (%) or mean (SD) as appropriate. Post hoc analyses assessed the difference in 3-month self-reported abstinence, smoking reduction, and quit attempts in the intervention group among those who had ever viewed the AR PHWs and those who had not. All analyses were conducted in Stata/MP (version 15.1; StataCorp). A 2-sided *P* value <.05 was considered statistically significant.

## Results

**Figure 2:**
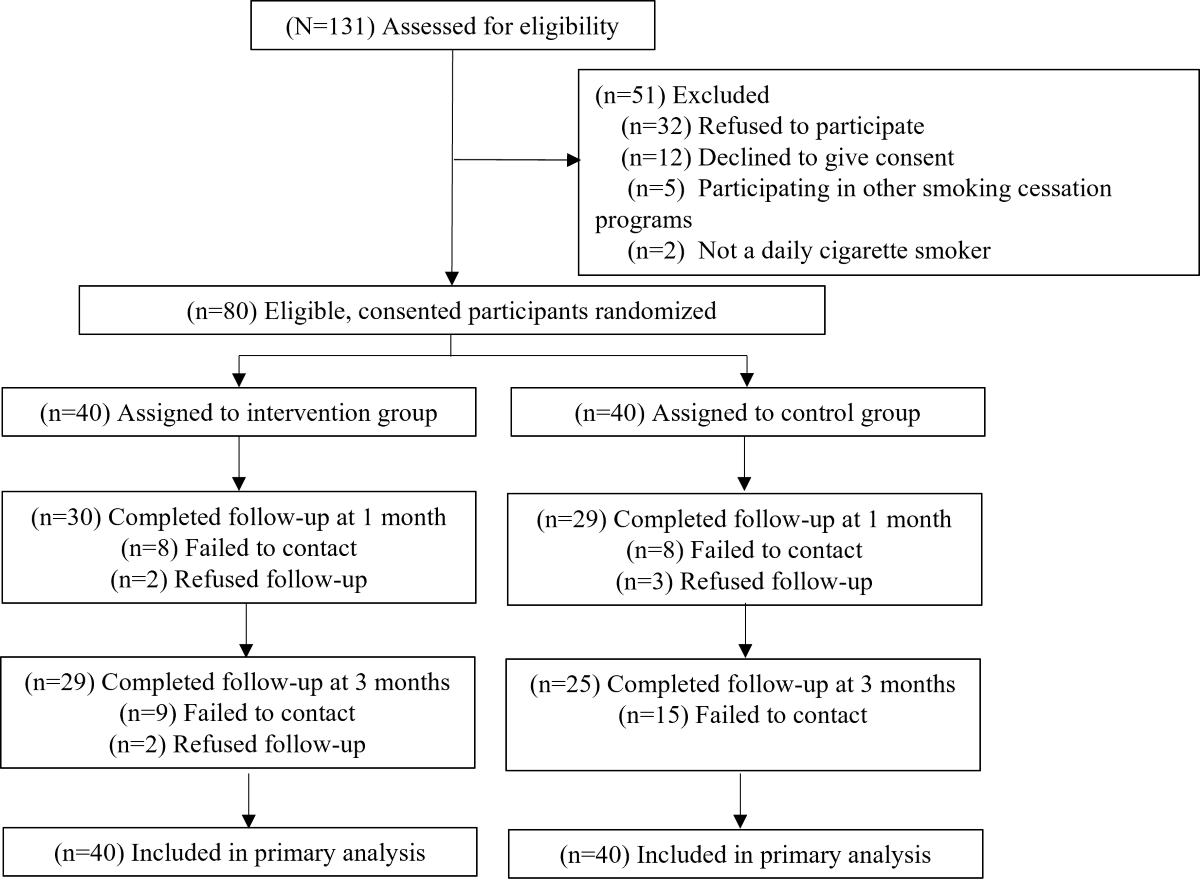
Trial flow chart.

Eighty participants were randomized to the intervention (n=40) and control (n=40) groups (Figure 2). Table 1 shows that 83% (66/80) of participants were male, 33% (26/80) were aged 50 years or above, and 65% (52/80) had secondary education or less. At baseline, 65% (52/80) had a low level of nicotine dependence, and 81% (65/80) planned to quit beyond 30 days (or were undecided). Baseline characteristics were similar between the 2 groups, except that more participants in the intervention group had tertiary education (*P*=.005). The follow-up retention rates were 74% (n=59) at 1 month and 68% (n=54) at 3 months; this was similar between the groups (*P*=.15 and .90).

Table 2 shows that the intervention group had a slightly higher but not statistically significant self-reported 7-day PPA (7/40, 18% vs 5/40, 13%; RR 1.40, 95% CI 0.48-4.07) and quit attempts (15/40, 38% vs 11/40, 28%; RR 1.36, 95% CI 0.71-2.60) than the control group at 3 months. Similar outcomes were observed after adjusting for levels of education. Participants in the control group had a higher smoking reduction rate compared to participants in the intervention group after excluding quitters, but the difference was not statistically significant at 3 months (4/33, 12% vs 8/35, 22%; RR 0.50, 95% CI 0.16-1.54).

Table 3 shows that 17 of 40 (43%) participants in the intervention group viewed the AR PHWs. Participants who viewed the AR PHWs rated them as having a mild effect on knowing the adverse consequences of smoking on personal health (mean rating 3.94, SD 3.52), reducing the frequency of buying cigarettes (mean rating 3.29, SD 3.08), perceiving an increase in the importance of quitting (mean rating 3.88, SD 3.50), finding PHWs more disgusting (mean rating 3.41, SD 3.08), and finding PHWs more horrible (mean rating 3.38, SD 3.05).

Table 4 shows the results from a post hoc analysis on viewing AR pictures and SC outcomes in the intervention group. The 3-month self-reported 7-day PPA was higher in those who ever (vs never) viewed AR PHWs (5/17, 29% vs 2/23, 9%). Similar results were observed for quit attempts at 3 months (8/17, 47% vs 7/23, 30%). The participants’ characteristics were similar regardless of ever viewing the AR PHWs (Multimedia Appendix 2).

**Table 1:** Participants’ characteristics at baseline (n=80).

Characteristics		Intervention group (n=40)	Control group (n=40)	*P* value^a^	
**Sex, n (%)**					.24
	Male	31 (78)	35 (88)		
	Female	9 (23)	5 (13)		
**Age (years), n (%)**					.78
	18-29	8 (20)	5 (13)		
	30-39	9 (23)	9 (23)		
	40-49	10 (25)	13 (33)		
	≥50	13 (33)	13 (33)		
**Education, n (%)**					.005
	Secondary or below	20 (50)	32 (80)		
	Tertiary	20 (50)	8 (20)		
**Monthly household income (US $1=HK $7.80), n (%)**					.26
	≤$19,999	7 (26)	13 (41)		
	$20,000-$29,999	5 (19)	8 (25)		
	>$30,000	15 (56)	11 (34)		
**Employment^b^, n (%)**					>.99
	Economically inactive	4 (10)	3 (8)		
	Economically active	36 (90)	37 (93)		
**Marital status, n (%)**					.88
	Single	16 (40)	15 (38)		
	Married	21 (53)	23 (58)		
	Divorced or widowed	3 (8)	2 (5)		
**Living with a child younger than 18 years, n (%)**					.48
	No	28 (70)	25 (63)		
	Yes	12 (30)	15 (38)		
Daily cigarette consumption, mean (SD)		12.31 (7.10)	11.31 (6.50)	.51	
**Nicotine dependency level^c^, n (%)**					.82
	Light	25 (63)	27 (68)		
	Moderate	14 (35)	13 (33)		
	Heavy	1 (3)	0 (0)		
**Past quit attempt, n (%)**					.17
	Within past 1 month	1 (3)	5 (13)		
	Within past 6 months	12 (30)	5 (13)		
	Within past 1 year	1 (3)	3 (8)		
	More than 1 year	12 (30)	12 (30)		
	Never	14 (35)	15 (38)		
**Willingness to quit, n (%)**					.24
	Within 7 days	4 (10)	6 (15)		
	Within 30 days	1 (3)	4 (10)		
	Within 60 days	2 (5)	1 (3)		
	Within 6 months	3 (8)	0 (0)		
	Undetermined	30 (75)	29 (73)		
**Self-efficacy, mean (SD)^d^, n (%)**					
	Perceived confidence of quitting	5.30 (2.57)	5.43 (2.22)	.82	
	Perceived difficulty of quitting	6.60 (2.31)	6.60 (2.04)	>.99	
	Perceived importance of quitting	4.83 (2.71)	5.13 (2.27)	.59	

^a^*P* values were calculated with the *χ*^2^ test or Fisher exact test for categorical variables and a 2-tailed *t* test for continuous variables.

^b^Economically active: employed or self-employed; economically inactive: student, housekeeper, retired, or unemployed.

^c^Measured by the Heaviness of Smoking Index (HSI): HSI score ≤2=light; HSI score 3-4=moderate; HSI score 5-6=heavy.

^d^Score range 0-10; higher scores indicate higher perceived importance, confidence, and difficulty of quitting.

**Table 2:** Smoking cessation outcomes in intervention vs control groups (N=80)^a^.

		Intervention (n=40), n (%)	Control (n=40), n (%)	Cohen ω	Risk ratio (95% CI)	*P* value	Adjusted risk ratio^b^ (95% CI)	*P* value
**Self-reported PPA^c^**								
	1 month	1 (3)	3 (8)	0.11	0.33 (0.04-3.11)	.34	0.35 (0.06-2.03)	.24
	3 month	7 (18)	5 (13)	0.07	1.40 (0.48-4.07)	.54	1.17 (0.40-3.39)	.78
**Smoking reduction^d^**								
	1 month	3 (8)	5 (14)	0.08	0.60 (0.15-2.36)	.47	0.80 (0.22-2.95)	.74
	3 month	4 (12)	8 (22)	0.14	0.50 (0.16-1.54)	.23	0.47 (0.14-1.58)	.22
**Quitting attempts**								
	1 month	10 (25)	11 (28)	0.06	0.91 (0.43-1.91)	.80	0.92 (0.42-2.00)	.84
	3 month	15 (38)	11 (28)	0.91	1.36 (0.71-2.60)	.35	1.30 (0.65-2.48)	.49

^a^Missing observations were treated as not quitting or reducing.

^a^Adjusted for education.

^c^PPA: point-prevalence abstinence.

^d^At least a 50% reduction in baseline daily cigarette consumption; participants who self-reported quitting were excluded.

**Table 3:** Viewing augmented reality (AR) pictures during the intervention period and perceptions of AR pictures (n=40)^a^.

			Values
**Viewing AR pictures, n (%)**			
	Never viewed^a^	23 (58)	
	Viewed	17 (43)	
**“AR pictures can help to...” (ratings of perceptions), mean (SD)**			
	Know the adverse consequences of smoking on personal health	3.94 (3.52)	
	Increase the perceived importance of quitting	3.88 (3.50)	
	Reduce the frequency of buying cigarettes	3.29 (3.08)	
	Perceive pictorial health warnings as more disgusting	3.41 (3.08)	
	Perceive pictorial health warnings as more horrible	3.38 (3.05)	

^a^Missing observations were treated as never having viewed the AR pictures.

**Table 4:** Smoking cessation outcomes at 3 months by whether participants had viewed the augmented reality (AR) pictures during the intervention period in the intervention group (n=40)^a^.

	Never viewed AR pictures (n=23), n (%)	Viewed AR pictures (n=17), n (%)	*P* value
Self-reported PPA^b^	2 (9)	5 (29)	.11
Smoking reduction^c^	3 (13)	1 (6)	.43
Quit attempts	7 (30)	8 (47)	.28

^a^Missing observations were treated as never having viewed the AR pictures.

^b^PPA: point-prevalence abstinence.

^c^At least a 50% reduction in baseline daily cigarette consumption; participants who self-reported quitting were excluded.

## Discussion

### Principal Findings

This is the first pilot RCT of mobile-based AR PHWs for SC in community smokers, showing that this approach was feasible and increased self-reported abstinence and quit attempts when provided on top of chat-based SC support. The 3-month 7-day PPA in the intervention (18%) and control (13%) groups in this study was comparable to that (15%) in our previous chat-based intervention for SC in 1185 smokers [2]. Notably, a direct comparison is not appropriate because of differences in the participants’ characteristics and the intervention. Our study adds to the literature on the potential of AR for SC.

Despite most of the participants having low willingness to quit at baseline, nearly half (17/40, 43%) of the intervention group participants viewed the AR PHWs. This might be due to the attractiveness of the novel AR modality and demonstrates the feasibility and acceptability of delivering AR intervention through mobile phones to community smokers. AR can vividly show the progression of diseases caused by smoking in an immersive, interactive manner, which might improve viewers’ acceptance of the warning information. Future mobile health (mHealth) SC interventions can involve AR as one component to attract and engage participants; mHealth interventions have been reported to have suboptimal engagement [32,33]. Notably, our AR links required scanning 6 types of PHWs on cigarette packs to activate the augmentation. Cigarettes with 12 types of PHWs are displayed in retail stores randomly, and participants bought cigarettes according to their preferences. Each type of package thus had a set PHW, and PHWs from other cigarette packages were lacking, which might have led to some AR PHWs not being activated or viewed. Even though we supplemented the PHWs in the chat-based SC support to mitigate this, supplementation required another device to display these PHWs to enable the activation of the AR content. These factors might have lowered the use of AR in this study. Future web AR content can be further designed with a function to scan pictures stored in the gallery on the users’ phones to reduce the burden of requiring another device or picture.

Participants rated the effect of AR PHWs on reducing how many times they bought cigarettes and the effect of evoking disgust and horrible feelings as mild. Our prior territory-wide survey after the implementation of new and enlarged PHWs found that most current smokers in Hong Kong were not considering quitting (69%) or deferring smoking (89.2%) after noticing PHWs [21]. Local PHWs have not been changed since 2018, and the warning effect of PHWs may be further diminished as a result of repeated viewing [34]. This might have weakened the perceived effect of our local PHW-based AR content on SC and eliciting negative emotions. Further AR content design can be refined by visualizing other adverse consequences of smoking. Apart from showing the deterioration of the organs, which might be familiar to smokers, using AR to show the mechanisms of smoking-related diseases at the microscopic level can be evaluated further. Prior studies have suggested AR can increase antismoking message acceptance by enhancing spatial presence (eg, the feeling of being there) and negative emotions [9,35]. Audio effects (eg, sound and music) to enhance immersion should be included to enhance the effect of AR.

Despite a perceived mild effect, we still observed more smoking abstinence (29% vs 9%) and quit attempts (47% vs 30%) in participants who viewed the AR PHWs compared to those who never did so. Most smokers understand the hazards of smoking but feel self-exemption from the adverse consequences of smoking [8,36]. Our AR was tailored to smoking behaviors, and the participants perceived relatively greater effects on understanding the adverse consequences of smoking on personal health and increasing the perceived importance of quitting. The AR PHWs might help participants connect adverse consequences of smoking with their own smoking behaviors to facilitate quitting.

Our finding of higher, but not statistically significant, smoking abstinence and quit attempts associated with the AR links combined with chat-based SC support could be due to a small sample size, which reduces the power to detect a statistically significant difference [37]. Since community smokers who are unmotivated to quit are less likely to download an SC app [38], we delivered AR links using instant messaging apps. The control group was offered chat-based SC support with similar content and intensity and a similar platform as the attention control. Since chat-based SC support is not usual care and may increase abstinence [2], adopting a strong effectiveness comparison may lead to underestimating the effect of AR PHWs on SC in the real world.

### Limitations

This study has some limitations. First, some baseline characteristics were imbalanced, which is common in small-sample trials [22]. Similar results were also observed after adjusting for the imbalances. Second, although a previous study has shown that a proactive recruitment approach can recruit smokers with demographic and smoking characteristics that are similar to those of general smokers in Hong Kong [39], the participants in this study were younger and more likely to have ever attempted to quit than general smokers (Multimedia Appendix 3). This difference, which might be due to the small sample size in this study, lowers the generalizability of the study findings. Additionally, most participants were male, which was in line with the sex distribution of smokers in Hong Kong [14]. Whether our findings can be generalized to other populations with more female smokers is uncertain. Third, abstinence may have been overreported without biochemical validation. But self-reports of abstinence were more feasible to obtain during the COVID-19 pandemic, and overreporting should have been similar in both groups. Fourth, this pilot study was underpowered to detect the effectiveness of mHealth-based AR PHWs on SC, and although our analysis of the intervention effect on smoking abstinence can be used to generate estimators for a full trial, our results cannot be interpreted as evidence for clinical practice. Fifth, data on the average number of AR PHWs each participant ever viewed were lacking, which might impede the exploration of the association between the dose of AR PHWs and SC. Sixth, the development of the AR PHWs did not involve smokers, which might affect the perception of the AR content in the intervention and its utility. Posttrial qualitative interviews are warranted to refine the AR content design.

### Implications

Considering that previous AR interventions for SC have always adopted a face-to-face approach [13], our findings provide initial evidence to support the feasibility of delivering AR interventions with a mobile modality. If the fully powered trial confirms the effect of the refined AR PHWs on smoking abstinence, mobile-based AR PHWs could be implemented to intervene in smoking behaviors. With the high accessibility of mobile phones, mobile-based AR interventions have the potential to be scalable in the real world, even in remote and resource-poor settings. These findings could also inform the development of mobile-based AR interventions for other harmful behaviors.

### Conclusion

We showed the initial feasibility of mobile-based interactive AR content and preliminary evidence on its effectiveness on smoking abstinence. Further AR interventions can consider enhancing participants’ immersive experiences and showing other adverse smoking-related consequences to maximize the effect on SC.

## Appendix

Multimedia Appendix 1 Six pictorial health warnings on cigarette packs in Hong Kong.

Multimedia Appendix 2 Characteristics of intervention group participants divided by whether they viewed the augmented reality pictorial health warnings (n=40).

Multimedia Appendix 3 Comparisons of the characteristics of the participants in this study and general smokers in Hong Kong.

Multimedia Appendix 4 CONSORT-eHEALTH checklist (V 1.6.1).

## Abbreviations

AR: augmented reality
AWARD: ask, warn, advise, referral, do-it-again
CONSORT: Consolidated Standards of Reporting Trials
HSI: Heaviness of Smoking Index
IM: instant messaging
mHealth: mobile health
MI: motivational interviewing
PHW: pictorial health warning
PPA: point-prevalence abstinence
RCT: randomized controlled trial
RR: risk ratio
SC: smoking cessation

## Data Availability

The data sets in this study are available from the corresponding author on reasonable request.
