# Supplementary figures and images for: Mobile Phone–Based Personalized and Interactive Augmented Reality Pictorial Health Warnings for Enhancing a Brief Advice Model for Smoking Cessation: Pilot Randomized Controlled Trial
Source: JMIR XR Spat Comput. 2024 Aug 1;1:e52893. doi: 10.2196/52893 (PMC13179107; doi:10.2196/52893)

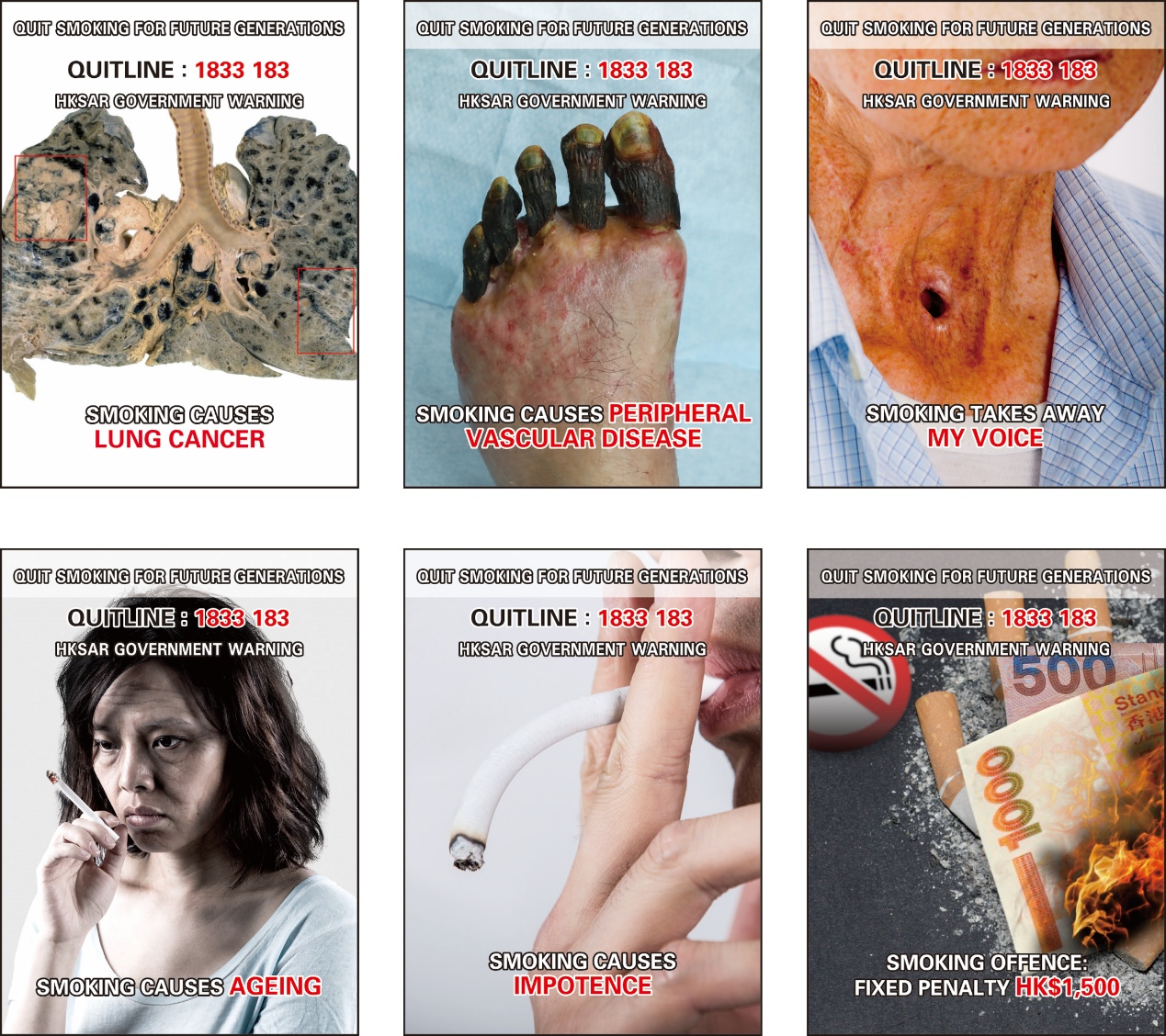


Multimedia Appendix 1. Six pictorial health warnings on cigarette packs in Hong Kong.

Supplement: Multimedia Appendix 1 [file xr_v1i1e52893_app1.docx]
