# Supplementary material for: Mobile Phone–Based Personalized and Interactive Augmented Reality Pictorial Health Warnings for Enhancing a Brief Advice Model for Smoking Cessation: Pilot Randomized Controlled Trial
Source: JMIR XR Spat Comput. 2024 Aug 1;1:e52893. doi: 10.2196/52893 (PMC13179107; doi:10.2196/52893)
Supplement: Multimedia Appendix 2 [file xr_v1i1e52893_app2.docx]

Multimedia Appendix 2. Intervention group participants characteristics by whether viewing AR pictures (N=40).

|  | Viewed AR pictures | Never viewed AR pictures | P value |
| --- | --- | --- | --- |
|  |  |  |  |
| **Sex** |  |  | 1.00 |
| Male | 13 (76.5) | 18 (78.3) |  |
| Female | 4 (23.5) | 5 (21.7) |  |
| **Education** |  |  | 0.75 |
| Secondary or below | 8 (47.1) | 12 (52.2) |  |
| Tertiary | 9 (52.9) | 11 (47.8) |  |
| **Income (HK$) ^a^** |  |  | 0.09 |
| ≤19999 | 1 (8.3) | 6 (40.0) |  |
| 20000-29999 | 4 (33.3) | 1 (6.7) |  |
| >30000 | 7 (58.3) | 8 (53.3) |  |
| **Nicotine dependency ^b^** |  |  | 0.25 |
| Light | 12 (70.6) | 13 (56.5) |  |
| Moderate | 4 (23.5) | 10 (43.5) |  |
| Heavy | 1 (5.9) | 0 (0.0) |  |
| **Quit attempt** |  |  | 0.93 |
| Within past 1 month | 1 (5.9) | 0 (0.0) |  |
| Within past 6 months | 5 (29.4) | 7 (30.4) |  |
| Within past 1 year | 0 (0.0) | 1 (4.4) |  |
| More than 1 year | 5 (29.4) | 7 (30.4) |  |
| Never | 6 (35.3) | 8 (34.8) |  |
| **Intention to quit** |  |  | 0.55 |
| Within 7 days | 2 (11.8) | 2 (8.7) |  |
| Within 30 days | 1 (5.9) | 0 (0.0) |  |
| Within 60 days | 0 (0.0) | 2 (8.7) |  |
| Within 6 months | 2 (11.8) | 1 (4.4) |  |
| Undetermined | 12 (70.6) | 18 (78.3) |  |

P value calculated by Chi-squared test or Fisher’s exact test.

^a^ US $1=HK$ 7.8

^b^ Measured by Heaviness of Smoking Index (HSI), the score of HSI ≤ 2 was light, the HSI score of 3-4 was moderate, the HSI score of 5-6 was heavy.
