# Supplementary material for: Mobile Phone–Based Personalized and Interactive Augmented Reality Pictorial Health Warnings for Enhancing a Brief Advice Model for Smoking Cessation: Pilot Randomized Controlled Trial
Source: JMIR XR Spat Comput. 2024 Aug 1;1:e52893. doi: 10.2196/52893 (PMC13179107; doi:10.2196/52893)
Supplement: Multimedia Appendix 3 [file xr_v1i1e52893_app3.docx]

Multimedia Appendix 3. Comparisons of characteristics between present study and general smokers in Hong Kong.

|  |  | Present study | General smokers ^a^ |
| --- | --- | --- | --- |
|  |  | N (%) | N (%) |
| Sex | |  |  |
|  | Male | 83 | 83.1 |
|  | Female | 18 | 16.9 |
| Age (years) | |  |  |
|  | 18-29 | 16 | 7.8 |
|  | 30-39 | 23 | 16.1 |
|  | 40-49 | 29 | 25.1 |
|  | ≥50 | 33 | 50.6 |
| Cigarette per day, mean | | 12 | 12.7 |
| Previous quit attempt | |  |  |
|  | No | 36 | 68.5 |
|  | Ever | 64 | 31.5 |

^a^ Data from Thematic Household Survey Report No. 75: Internet and personal computer penetration & Pattern of smoking.
