# Supplementary material for: Mobile Phone–Based Personalized and Interactive Augmented Reality Pictorial Health Warnings for Enhancing a Brief Advice Model for Smoking Cessation: Pilot Randomized Controlled Trial
Source: JMIR XR Spat Comput. 2024 Aug 1;1:e52893. doi: 10.2196/52893 (PMC13179107; doi:10.2196/52893)
Supplement: Multimedia Appendix 4 [file xr_v1i1e52893_app4.pdf]

CONSORT-EHEALTH Checklist V1.6.2 Report	Manuscript Number	52893
**(based on CONSORT-EHEALTH V1.6), available at [http://tinyurl.com/consort-ehealth-v1-6].**		
**Date completed**		
8/1/2024 2:41:48		
**by**		
Ziqiu Guo		
Mobile Phone–Based Personalized and Interactive Augmented Reality Pictorial Health Warnings for Enhancing a Brief Advice Model for Smoking Cessation: Pilot Randomized Controlled Trial		
TITLE		
**1a-i) Identify the mode of delivery in the title**		
"Mobile Phone–Based Personalized and Interactive Augmented Reality Pictorial Health Warnings for Enhancing a Brief Advice Model for Smoking Cessation: Pilot Randomized Controlled Trial"		
**1a-ii) Non-web-based components or important co-interventions in title**		
"Mobile Phone–Based Personalized and Interactive Augmented Reality Pictorial Health Warnings for Enhancing a Brief Advice Model for Smoking Cessation: Pilot Randomized Controlled Trial"		
**1a-iii) Primary condition or target group in the title**		
We have indicated that the trial was for smoking cessation, which can imply that our study was targeted at smokers.		
ABSTRACT		
**1b-i) Key features/functionalities/components of the intervention and comparator in the METHODS section of the ABSTRACT**		
"All participants received AWARD (ask, warn, advise, referral, do-it-again) model–guided SC advice, a warning leaflet, and referral to SC services at baseline. Interactive, chat-based SC support comprising regular messages and real-time support was provided to all participants via instant messaging apps (eg, WhatsApp) for 3 months after randomization. Participants in the intervention group additionally received 6 links to the AR PHWs showing the worsening health status of various organs caused by smoking. The level of the AR PHWs was adjustable to smoking behaviors (ie, smoking duration or daily cigarette consumption) to increase interaction. Participants could swipe, drag, or rotate the 3D PHWs to reinforce their impression of the health consequences of smoking. "		
**1b-ii) Level of human involvement in the METHODS section of the ABSTRACT**		
We did not clarify the level of human involvement in the abstract since the study was aimed to evaluate the AR pictorial health warning.		
**1b-iii) Open vs. closed, web-based (self-assessment) vs. face-to-face assessments in the METHODS section of the ABSTRACT**		
We did not report this in the abstract but in the main body of the text.		
**1b-iv) RESULTS section in abstract must contain use data**		
"In the intervention group, 17 of 40 (43%) participants viewed the AR PHWs."		
**1b-v) CONCLUSIONS/DISCUSSION in abstract for negative trials**		
"The mobile-based interactive AR PHWs were feasible, and the effectiveness on smoking abstinence warrants further testing."		
INTRODUCTION		
**2a-i) Problem and the type of system/solution**		
"This study used the notion that PHWs are widely viewed by the public on cigarette packaging and developed 6 new, localized, AR-based PHWs (Multimedia Appendix 1). Taking advantage of IM apps, this pilot study aimed to examine the feasibility and preliminary effect of AR PHWs on SC."		
**2a-ii) Scientific background, rationale: What is known about the (type of) system**		
"AR-simulated adverse consequences of smoking (eg, in the lungs) were used in educating nonsmokers about smoking hazards to the human body [10] and dissuading nonsmokers from smoking [11], but the effects were not assessed using a rigorous research design. Recent studies have suggested the potential of AR as a novel modality for conducting cue exposure therapy for SC (ie, extinguishing cue-provoked urges to smoke by exposure to smoking-related cues) since smoking-related AR images were found to have a similar effect on eliciting cue-provoked urges to smoke compared to in vivo smoking cues [12,13]. Our literature review of PubMed (up to September 2023) using the terms “augmented reality” and “smoking cessation” found no study assessing the effect of AR visualization of adverse consequences from smoking on SC. "		
**Does your paper address CONSORT subitem 2b?**		
"Taking advantage of IM apps, this pilot study aimed to examine the feasibility and preliminary effect of AR PHWs on SC." No hypotheses for the pilot study.		
METHODS		
**3a) CONSORT: Description of trial design (such as parallel, factorial) including allocation ratio**		
"We conducted a parallel, 2-arm, pilot randomized controlled trial (RCT) in Hong Kong."		
**3b) CONSORT: Important changes to methods after trial commencement (such as eligibility criteria), with reasons**		
No changes have been made after trial commencement.		
**3b-i) Bug fixes, Downtimes, Content Changes**		
There are no bug fixes for the AR pictorial health warnings. Since this was a pilot study, we explored the participants' perceptions of the AR pictorial health warnings and discussed further revisions in the discussion section.		
**4a) CONSORT: Eligibility criteria for participants**		
"Eligibility criteria included being Chinese, being a smoker, being aged 18 years or older, smoking at least 1 cigarette daily in the past 30 days, owning a smartphone with IM apps (eg, WhatsApp), and being able to communicate in Chinese. We excluded smokers who were participating in other SC services or projects."		
**4a-i) Computer / Internet literacy**		
This study did not consider the eHealth literacy level since the mobile-based intervention was directly sent to participants via commonly used instant messaging apps. The intervention content also was designed straightforwardly. We admitted that the eHealth literacy level might have affected the understanding of the intervention, but participants were randomly assigned to the intervention and control groups; this might have balanced the potential effects of different eHealth literacy levels.		
**4a-ii) Open vs. closed, web-based vs. face-to-face assessments:**		
"From April to November 2021, participants were individually recruited at smoking hot spots (ie, places where smokers linger and smoke) in community sites. Research staff proactively approached smokers using a “foot-in-the-door” technique, which is commonly used in SC trials in the context of smokers with low intention to quit [1,2,23] Briefly, the research staff initiated a conversation by asking about small and simple questions like their daily cigarette consumption and quit attempts. Those willing to talk were warned about the risk of continuous smoking and advised to quit smoking as early as possible. Smokers who expressed interest in SC were briefly introduced to the trial, screened for eligibility, and invited to join."		
**4a-iii) Information giving during recruitment**		
"Briefly, the research staff initiated a conversation by asking about small and simple questions like their daily cigarette consumption and quit attempts. Those willing to talk were warned about the risk of continuous smoking and advised to quit smoking as early as possible. Smokers who expressed interest in SC were briefly introduced to the trial, screened for eligibility, and invited to join. " ... "Written consent was obtained from all participants after informing them that they had the right to withdraw from the study at any time, and all the information collected was anonymized and was only used for research. After completing each follow-up, all participants were compensated with HK$ 50 (US$ 6.40)."		
**4b) CONSORT: Settings and locations where the data were collected**		
The data were collected at community setting in Hong Kong. "We conducted a parallel, 2-arm, pilot randomized controlled trial (RCT) in Hong Kong. From April to November 2021, participants were individually recruited at smoking hot spots (ie, places where smokers linger and smoke) in community sites. "		
**4b-i) Report if outcomes were (self-)assessed through online questionnaires**		
Telephone follow-ups were conducted at 1 month and 3 months after intervention initiation.		
**4b-ii) Report how institutional affiliations are displayed**		
**5) CONSORT: Describe the interventions for each group with sufficient details to allow replication, including how and when they were actually administered**		
**5-i) Mention names, credential, affiliations of the developers, sponsors, and owners**		

"One AR technology company developed the AR links with the SC experts' continuous comments on the shape and color of the models and necessary changes. " ... "We thank the participants for their responses to the study and Softmind Ltd for building the augmented reality content. This work was supported by the Seed Fund for Basic Research of the University of Hong Kong."		
**5-ii) Describe the history/development process**		
This was the first time we developed and evaluated the AR pictorial health warnings.		
**5-iii) Revisions and updating**		
This pilot study aimed to explore the acceptability of AR pictorial health warnings. Therefore, these AR warnings were frozen during the trial and would be updated based on the study findings.		
**5-iv) Quality assurance methods**		
"Guided by motivational interviewing (MI) techniques [25] and the transtheoretical model [26], the chat-based SC support included prescheduled messages and real-time support personalized to the quitting process provided by 1 SC advisor (a registered nurse trained in SC treatments and MI techniques). " ... "The SC advisor discussed the supporting content with experts in the SC team when necessary."		
**5-v) Ensure replicability by publishing the source code, and/or providing screenshots/screen-capture video, and/or providing flowcharts of the algorithms used**		
"the AR PHW for "smoking causes peripheral vascular disease" is shown in Figure 1"		
**5-vi) Digital preservation**		
The intervention content can only be accessed by specific groups.		
**5-vii) Access**		
"As a widely adopted alternative practice for smokers who are less ready to quit in SC programs [2,24], chat-based SC support was delivered to all participants for 3 months via IM apps (ie, WhatsApp)." ... "Six web AR links were integrated into the messages during the chat-based SC support for the intervention group."		
**5-viii) Mode of delivery, features/functionalities/components of the intervention and comparator, and the theoretical framework**		
"Guided by motivational interviewing (MI) techniques [25] and the transtheoretical model [26], the chat-based SC support included prescheduled messages and real-time support personalized to the quitting process provided by 1 SC advisor (a registered nurse trained in SC treatments and MI techniques)." ... "One AR technology company developed the AR links with the SC experts' continuous comments on the shape and color of the models and necessary changes. All the AR PHWs were designed to increase the participants' awareness of the devastating effects on health of smoking and its ever-growing monetary cost, thereby promoting their engagement in the chat-based SC support."		
**5-ix) Describe use parameters**		
"Table 3 shows that 17 of 40 (43%) participants in the intervention group viewed the AR PHWs." ... "Fifth, data on the average number of AR PHWs each participant ever viewed were lacking, which might impede the exploration of the association between the dose of AR PHWs and SC."		
**5-x) Clarify the level of human involvement**		
"As an alternative way to access the AR PHWs, we provided the PHW images on a separate device (eg, a personal computer running WhatsApp) so that the users could scan the images with their mobile phones."		
**5-xi) Report any prompts/reminders used**		
"At least 1 message was delivered per week to initiate the conversation between participants and the SC advisor." ... "The SC advisor also encouraged participants to click the links and interact with the AR PHWs and supplemented the information (eg, with corresponding PHWs or a brief introduction) as appropriate."		
**5-xii) Describe any co-interventions (incl. training/support)**		
"All participants received brief SC advice following the AWARD (ask, warn, advise, referral, do-it-again) model [1-3] and were offered referrals to SC services at baseline."		
**6a) CONSORT: Completely defined pre-specified primary and secondary outcome measures, including how and when they were assessed**		
"The primary outcome was self-reported 7-day point-prevalence abstinence (PPA) at the 3-month follow-up. Secondary outcomes included the 7-day PPA at the 1-month follow-up, smoking reduction by at least 50% of baseline daily cigarette consumption (excluding quitters), and quit attempts (defined by abstinence for at least 24 hours) at the 1- and 3-month follow-ups" ... "Considering that proactive recruitment and chat-based support via IM apps have shown feasibility in our previous SC studies [1,2,17,28], we calculated the proportion of participants who had viewed the AR PHWs during the intervention to assess the acceptability of the AR intervention component. The proportion was defined as the actual number of participants who reported having ever viewed the AR PHWs at the follow-ups divided by the number of participants in the intervention group. Missing values were treated as "no viewing.""		
**6a-i) Online questionnaires: describe if they were validated for online use and apply CHERRIES items to describe how the questionnaires were designed/deployed**		
The outcomes were measured by telephone interviews.		
**6a-ii) Describe whether and how "use" (including intensity of use/dosage) was defined/measured/monitored**		
"Considering that proactive recruitment and chat-based support via IM apps have shown feasibility in our previous SC studies [1,2,17,28], we calculated the proportion of participants who had viewed the AR PHWs during the intervention to assess the acceptability of the AR intervention component. The proportion was defined as the actual number of participants who reported having ever viewed the AR PHWs at the follow-ups divided by the number of participants in the intervention group. Missing values were treated as "no viewing.""		
**6a-iii) Describe whether, how, and when qualitative feedback from participants was obtained**		
We did not collect qualitative feedback in this study.		
**6b) CONSORT: Any changes to trial outcomes after the trial commenced, with reasons**		
The data were collected at community setting in Hong Kong. "We conducted a parallel, 2-arm, pilot randomized controlled trial (RCT) in Hong Kong. From April to November 2021, participants were individually recruited at smoking hot spots (ie, places where smokers linger and smoke) in community sites. "		
**7a) CONSORT: How sample size was determined**		
**7a-i) Describe whether and how expected attrition was taken into account when calculating the sample size**		
"No formal sample size calculation was conducted. The sample size of 80 participants is comparable with previous studies of virtual reality interventions (ranging from 8-102 [29]) for SC and was deemed sufficient to test the feasibility and estimate the parameters to inform the design of a definitive RCT."		
**7b) CONSORT: When applicable, explanation of any interim analyses and stopping guidelines**		
"The primary outcome was self-reported 7-day point-prevalence abstinence (PPA) at the 3-month follow-up. Secondary outcomes included the 7-day PPA at the 1-month follow-up, smoking reduction by at least 50% of baseline daily cigarette consumption (excluding quitters), and quit attempts (defined by abstinence for at least 24 hours) at the 1- and 3-month follow-ups" ... "Considering that proactive recruitment and chat-based support via IM apps have shown feasibility in our previous SC studies [1,2,17,28], we calculated the proportion of participants who had viewed the AR PHWs during the intervention to assess the acceptability of the AR intervention component. The proportion was defined as the actual number of participants who reported having ever viewed the AR PHWs at the follow-ups divided by the number of participants in the intervention group. Missing values were treated as "no viewing.""		
**8a) CONSORT: Method used to generate the random allocation sequence**		
"Participants were individually randomized to intervention or control groups (at a 1:1 ratio) according to a predefined randomization list with permuted block sizes of 2, 4, and 6. The allocation sequence was generated by a noninvestigator and was concealed from the research staff involved in participant recruitment. "		
**8b) CONSORT: Type of randomisation; details of any restriction (such as blocking and block size)**		
"Participants were individually randomized to intervention or control groups (at a 1:1 ratio) according to a predefined randomization list with permuted block sizes of 2, 4, and 6. "		
**9) CONSORT: Mechanism used to implement the random allocation sequence (such as sequentially numbered containers), describing any steps taken to conceal the sequence until interventions were assigned**		
"Blinding of participants and the SC advisor who delivered the interventions was not possible. All outcome assessors and statistical analysts were blinded to treatment allocation until the primary analyses were completed."		
**10) CONSORT: Who generated the random allocation sequence, who enrolled participants, and who assigned participants to interventions**		
"The allocation sequence was generated by a noninvestigator and was concealed from the research staff involved in participant recruitment." ... "Research staff proactively approached smokers using a "foot-in-the-door" technique, which is commonly used in SC trials in the context of smokers with low intention to quit [1,2,23] "		
**11a) CONSORT: Blinding - If done, who was blinded after assignment to interventions (for example, participants, care providers, those assessing outcomes) and how**		
**11a-i) Specify who was blinded, and who wasn't**		
"Blinding of participants and the SC advisor who delivered the interventions was not possible. All outcome assessors and statistical analysts were blinded to treatment allocation until the primary analyses were completed."		
**11a-ii) Discuss e.g., whether participants knew which intervention was the "intervention of interest" and which one was the "comparator"**		

"Blinding of participants and the SC advisor who delivered the interventions was not possible."		
**11b) CONSORT: If relevant, description of the similarity of interventions**		
Participants in the control group also received AWARD advice, referral to smoking cessation services, and chat-based support.		
**12a) CONSORT: Statistical methods used to compare groups for primary and secondary outcomes**		
"We compared the primary and secondary outcomes between the 2 groups using Poisson regression with robust variance [30], which yielded risk ratios (RRs) of the intervention effect."		
**12a-i) Imputation techniques to deal with attrition / missing values**		
"The intention to treat approach was adopted, assuming participants lost to follow-up did not change their smoking behaviors (eg, they made no quit attempts nor changed their daily cigarette consumption) [31]."		
**12b) CONSORT: Methods for additional analyses, such as subgroup analyses and adjusted analyses**		
"Post hoc analyses assessed the difference in 3-month self-reported abstinence, smoking reduction, and quit attempts in the intervention group among those who had ever viewed the AR PHWs and those who had not."		
RESULTS		
**13a) CONSORT: For each group, the numbers of participants who were randomly assigned, received intended treatment, and were analysed for the primary outcome**		
"Eighty participants were randomized to the intervention (n=40) and control (n=40) groups (Figure 2)." and Figure 2		
**13b) CONSORT: For each group, losses and exclusions after randomisation, together with reasons**		
Figure 2 is a CONSORT flow diagram.		
**13b-i) Attrition diagram**		
The intervention lasted 3 months, and participants were followed for 3 months. We did not collect data on using the intervention after the study finished.		
**14a) CONSORT: Dates defining the periods of recruitment and follow-up**		
"From April to November 2021, participants were individually recruited at smoking hot spots (ie, places where smokers linger and smoke) in community sites." ... "Telephone follow-ups were conducted at 1 month and 3 months after intervention initiation."		
**14a-i) Indicate if critical "secular events" fell into the study period**		
No secular events fell into the study period.		
**14b) CONSORT: Why the trial ended or was stopped (early)**		
The trial was ended according to the proposal.		
**15) CONSORT: A table showing baseline demographic and clinical characteristics for each group**		
Table 1 showed the baseline characteristics for each group.		
**15-i) Report demographics associated with digital divide issues**		
"The participants' characteristics were similar regardless of ever viewing the AR PHWs (Multimedia Appendix 2)."		
**16a) CONSORT: For each group, number of participants (denominator) included in each analysis and whether the analysis was by original assigned groups**		
**16-i) Report multiple "denominators" and provide definitions**		
"Table 1 shows that 83% (66/80) of participants were male, 33% (26/80) were aged 50 years or above, and 65% (52/80) had secondary education or less. At baseline, 65% (52/80) had a low level of nicotine dependence, and 81% (65/80) planned to quit beyond 30 days (or were undecided)."... "Table 3 shows that 17 of 40 (43%) participants in the intervention group viewed the AR PHWs." ... "The 3-month self-reported 7-day PPA was higher in those who ever (vs never) viewed AR PHWs (5/17, 29% vs 2/23, 9%). Similar results were observed for quit attempts at 3 months (8/17, 47% vs 7/23, 30%)."		
**16-ii) Primary analysis should be intent-to-treat**		
"The intention to treat approach was adopted, assuming participants lost to follow-up did not change their smoking behaviors (eg, they made no quit attempts nor changed their daily cigarette consumption) [31]. "		
**17a) CONSORT: For each primary and secondary outcome, results for each group, and the estimated effect size and its precision (such as 95% confidence interval)**		
"Table 2 shows that the intervention group had a slightly higher but not statistically significant self-reported 7-day PPA (7/40, 18% vs 5/40, 13%; RR 1.40, 95% CI 0.48-4.07) and quit attempts (15/40, 38% vs 11/40, 28%; RR 1.36, 95% CI 0.71-2.60) than the control group at 3 months."		
**17a-i) Presentation of process outcomes such as metrics of use and intensity of use**		
"Table 3 shows that 17 of 40 (43%) participants in the intervention group viewed the AR PHWs."		
**17b) CONSORT: For binary outcomes, presentation of both absolute and relative effect sizes is recommended**		
"Table 2 shows that the intervention group had a slightly higher but not statistically significant self-reported 7-day PPA (7/40, 18% vs 5/40, 13%; RR 1.40, 95% CI 0.48-4.07) and quit attempts (15/40, 38% vs 11/40, 28%; RR 1.36, 95% CI 0.71-2.60) than the control group at 3 months."		
**18) CONSORT: Results of any other analyses performed, including subgroup analyses and adjusted analyses, distinguishing pre-specified from exploratory**		
"Similar outcomes were observed after adjusting for levels of education."		
**18-i) Subgroup analysis of comparing only users**		
"The 3-month self-reported 7-day PPA was higher in those who ever (vs never) viewed AR PHWs (5/17, 29% vs 2/23, 9%). Similar results were observed for quit attempts at 3 months (8/17, 47% vs 7/23, 30%)."		
**19) CONSORT: All important harms or unintended effects in each group**		
The study was a mobile-based intervention; it contributed to smoking cessation and did not cause any harm.		
**19-i) Include privacy breaches, technical problems**		
The participants did not express any technical problems when opening the AR pictorial health warnings links.		
**19-ii) Include qualitative feedback from participants or observations from staff/researchers**		
We did not collect any qualitative data in the study and discussed this limitation in the discussion section.		
DISCUSSION		
**20) CONSORT: Trial limitations, addressing sources of potential bias, imprecision, multiplicity of analyses**		
**20-i) Typical limitations in ehealth trials**		
"Fifth, data on the average number of AR PHWs each participant ever viewed were lacking, which might impede the exploration of the association between the dose of AR PHWs and SC. Sixth, the development of the AR PHWs did not involve smokers, which might affect the perception of the AR content in the intervention and its utility. Posttrial qualitative interviews are warranted to refine the AR content design."		
**21) CONSORT: Generalisability (external validity, applicability) of the trial findings**		
**21-i) Generalizability to other populations**		
"Second, although a previous study has shown that a proactive recruitment approach can recruit smokers with demographic and smoking characteristics that are similar to those of general smokers in Hong Kong [39], the participants in this study were younger and more likely to have ever attempted to quit than general smokers (Multimedia Appendix 3). This difference, which might be due to the small sample size in this study, lowers the generalizability of the study findings. Additionally, most participants were male, which was in line with the sex distribution of smokers in Hong Kong [14]. Whether our findings can be generalized to other populations with more female smokers is uncertain."		
**21-ii) Discuss if there were elements in the RCT that would be different in a routine application setting**		
"Further AR content design can be refined by visualizing other adverse consequences of smoking. Apart from showing the deterioration of the organs, which might be familiar to smokers, using AR to show the mechanisms of smoking-related diseases at the microscopic level can be evaluated further. Prior studies have suggested AR can increase antismoking message acceptance by enhancing spatial presence (eg, the feeling of being there) and negative emotions [9,35]. Audio effects (eg, sound and music) to enhance immersion should be included to enhance the effect of AR."		
**22) CONSORT: Interpretation consistent with results, balancing benefits and harms, and considering other relevant evidence**		
**22-i) Restate study questions and summarize the answers suggested by the data, starting with primary outcomes and process outcomes (use)**		
"This is the first pilot RCT of mobile-based AR PHWs for SC in community smokers, showing that this approach was feasible and increased self-reported abstinence and quit attempts when provided on top of chat-based SC support. "		
**22-ii) Highlight unanswered new questions, suggest future research**		
"Further AR content design can be refined by visualizing other adverse consequences of smoking. Apart from showing the deterioration of the organs, which might be familiar to smokers, using AR to show the mechanisms of smoking-related diseases at the microscopic level can be evaluated further. Prior studies have suggested AR can increase antismoking message acceptance by enhancing spatial presence (eg, the feeling of being there) and negative emotions [9,35]. Audio effects (eg, sound and music) to enhance immersion should be included to enhance the effect of AR."		
Other information		

**23) CONSORT: Registration number and name of trial registry**		
"Trial Registration: ClinicalTrials.gov NCT04830072; https://clinicaltrials.gov/study/NCT04830072"		
**24) CONSORT: Where the full trial protocol can be accessed, if available**		
The full trial protocol has been submitted to the university grants committee.		
**25) CONSORT: Sources of funding and other support (such as supply of drugs), role of funders**		
"This work was supported by the Seed Fund for Basic Research of the University of Hong Kong."		
**X26-i) Comment on ethics committee approval**		
"Ethical approval was obtained from the institutional review board of the University of Hong Kong/Hospital Authority Hong Kong West Cluster (UW 21-018)"		
**x26-ii) Outline informed consent procedures**		
"Written consent was obtained from all participants after informing them that they had the right to withdraw from the study at any time, and all the information collected was anonymized and was only used for research. After completing each follow-up, all participants were compensated with HK$ 50 (US$ 6.40)."		
**X26-iii) Safety and security procedures**		
"Written consent was obtained from all participants after informing them that they had the right to withdraw from the study at any time, and all the information collected was anonymized and was only used for research."		
**X27-i) State the relation of the study team towards the system being evaluated**		
"Conflicts of Interest: None declared."		
